# Clinical and Organizational Impacts of Medical Ordering Settings on Patient Pathway and Community Pharmacy Dispensing Process: The Prospective ORDHOSPIVILLE Study

**DOI:** 10.3390/pharmacy10010002

**Published:** 2021-12-23

**Authors:** Justine Clarenne, Julien Gravoulet, Virginie Chopard, Julia Rouge, Amélie Lestrille, François Dupuis, Léa Aubert, Sophie Malblanc, Coralie Barbe, Florian Slimano, Céline Mongaret

**Affiliations:** 1 Faculty of Pharmacy, Université de Reims Champagne-Ardenne, 51 Rue Cognacq-Jay, 51100 Reims, France; lestrille.amelie@gmail.com (A.L.); laubert@outlook.fr (L.A.); florian.slimano@univ-reims.fr (F.S.); celine.mongaret@univ-reims.fr (C.M.); 2 Department of Pharmacy, CHU Reims, Rue du Général Koenig, 51100 Reims, France; 3 Faculty of Pharmacy, Université de Lorraine, 7 Avenue de la Forêt de Haye, 54500 Nancy, France; julien@gravoulet.fr (J.G.); francois.dupuis@univ-lorraine.fr (F.D.); 4 Pharmacie Gravoulet, 5 Rue du Haut Château, 54760 Leyr, France; 5 OMéDIT Grand Est, 3 Boulevard Joffre, 54000 Nancy, France; Virginie.chopard@ars.sante.fr (V.C.); rouge.julia@gmail.com (J.R.); 6 Agence Régionale de Santé, 3 Boulevard Joffre, 54000 Nancy, France; sophie.malblanc@ars.sante.fr; 7 Comité Universitaire de Ressources pour la Recherche en Santé, Pôle Santé, Université de Reims Champagne-Ardenne, 51 Rue Cognacq-Jay, 51100 Reims, France; coralie.barbe1@univ-reims.fr

**Keywords:** drug-related problems, community pharmacist, hospital discharge, pharmacist intervention, clinical impact, organizational impact

## Abstract

During the dispensing process of medical orders (MOs), community pharmacists (CPs) can manage drug-related problems (DRPs) by performing pharmacist interventions (PIs). There is little evidence that the PI rate is higher with MOs from hospitals (MOHs) than ambulatory (MOAs) settings, and their impact on the patient and community pharmacy is unknown. The primary objective of this study was to compare the MOH and MOA PI rates. The secondary objective was to describe PIs and their clinical and organizational impacts on patient and community pharmacy workflow. A total of 120 CPs participated in a prospective study. Each CP included 10 MOH and 10 MOA between January and June 2020. DRP and PI description and clinical and organizational impacts between MOH and MOA were assessed and compared. We analyzed 2325 MOs. PIs were significantly more frequent in MOH than in MOA (9.7% versus 4.7%; *p* < 0.001). The most reported PI was the difficulty of contacting hospital prescribers (n = 45; 52.2%). MOHs were associated with a longer dispensing process time and a greater impact on patient pathway and community pharmacy workflow than MOAs. Lack of communication between hospital and primary care settings partly explains the results. Implementation of clinical pharmacy activities at patient discharge could alleviate these impacts.

## 1. Introduction

A large part of the community pharmacist’s working time is devoted to the dispensation of medical orders (MOs) for all types of patients, mainly coming from ambulatory or hospital settings [1]. Community pharmacists (CPs) are typically the first healthcare professionals to interact with recently discharged patients, which is a high-risk step in patient care pathway [2] or after hospital consultation.

During the dispensation process, CPs may detect drug-related problems (DRPs) [3] that they must manage and track by performing pharmacist interventions (PIs). In France, the French Society of Clinical Pharmacy (SFPC) developed and validated a reporting tool for the uniform detection and classification of pharmacist interventions in our hospitals [4]. In 2018, this tool was adapted by a group of experts designed by the SFPC for use in community pharmacy settings [5]. DRPs and PIs can be classified into eleven and seven categories, respectively. Additional DRPs can be detected on hospital Mos for several reasons such as the time of discharge, infrequently used or very expensive drugs (most of them unavailable immediately), and difficulties in calling prescribers [6,7,8]. Each DRP can not only result in clinical impacts for the patient but also in organizational impacts for community pharmacy teams (e.g., additional workload and difficulties related to professional task shifting) and patients (e.g., additional visits to the pharmacy).

In France, medical orders from hospital are reputed to be at high risk of drug-related problems (DRPs), but this is only based on the general feeling of community pharmacists. Only one study by Michel et al. reported the prevalence of DRPs detected by community pharmacists when dispensing hospital discharge prescriptions [2]. However, they did not compare these results with the prevalence of DRPs on MOAs. In order to characterize the potential specificities of DRPs on MOHs, especially in terms of clinical and organizational impact, we designed a prospective study at the regional level.

The primary objective of this study was to compare the rate of PIs performed by CPs between MOs issued from hospital (MOHs) or from ambulatory settings (MOAs). Then, the potential clinical relevance of these PIs, their potential clinical impact on patient care pathway, and the organizational impact on CP workflow were evaluated.

## 2. Materials and Methods

### 2.1. Study Settings

This is a prospective study organized by the Faculties of Pharmacy of Nancy (Université de Lorraine) and Reims (Université de Reims Champagne-Ardenne). The study was conducted in 120 community pharmacies in the Grand Est region, France. The details of pharmacy location (city-center pharmacy, neighborhood pharmacy, rural pharmacy, or pharmacy in shopping center) were not precisely collected, but repartition was supposed to be comparable to other regions in France. All the participating pharmacies were independent (there are no community pharmacy chains in France) and welcomed a pharmacy student during their last 6-month internship [9] under supervision of their mentor pharmacists. All mentor pharmacists had graduated at least five years ago and completed updated training each year before the 6-month internship formation. The inclusion period of medical orders lasted 6 months from January to June 2020. Due to the fact that the dispensation of medical orders from hospital setting (MOHs) is less frequent than medical orders from ambulatory settings (MOAs), each pharmacist included the 10 first MOHs they dispensed. Each inclusion of a MOH immediately resulted in the inclusion of the consecutive MOA they experienced at the pharmacy. A total of 10 MOHs and 10 MOAs were included in pairs for each community pharmacy.

This study was conducted in compliance with French legislation related to observational studies. Patient consent was obtained for all the subjects included in the study. Patients’ records were anonymized prior to analysis. The database was constituted and collected in accordance with the reference methodology MR004 of the Commission Nationale de l’Informatique et des Libertés (no. 2212122, date: 3 July 2019). The study was declared at the national registry of health research (Health Data Hub) under number F20210907111038.

### 2.2. Inclusion and Noninclusion Criteria

Each medical order with at least one drug, medical device, and/or other health product was included. MOs from hospital settings (all types) were written after a medical consultation or hospital discharge (regardless of the hospitalization length). MOs from ambulatory settings could be written by a general practitioner or specialist.

Every MO with incomplete or duplicate electronic collection forms was excluded.

### 2.3. Data Recorded

During the dispensation process, pharmacists detected DRPs for each MOH and MOA and then performed PIs according to the tool from the French Society of Clinical Pharmacy (SFPC) [3]. The potential clinical impact of each PI was prospectively assessed using the CLEO© tool from the SFPC [10]. For each MO, the pharmacists completed an electronic collection form (Supplementary Material) including 3 parts: information about MO (nature of the MO, identification of the individual bringing the MO to the pharmacy, nature and identification of the prescriber, and number of prescribed medications including medical devices and others); PI description (drug, DRP, outcome of the PI, and the potential clinical impact); and assessment of pharmaceutical care. Clinical impact was classified into 6 categories from harmful outcome of the PI (-1C) to lethal outcome avoidance for the patient (4C). Finally, the impacts on the patient care pathway and on community pharmacy workflow were assessed by using the delay of availability of the prescribed medications and the organizational impact on the patient’s visit at the pharmacy.

### 2.4. Statistical Analysis

The chi-square test was used for the comparison of MOHs and MOAs. All statistical analyses were computed using R^®^ (The R Project for Statistical Computing v.3.2.2). A *p*-value less than 0.05 was considered statistically significant.

## 3. Results

A total of 2325 prescriptions were included in the study: 1151 MOAs (49.5%) and 1174 MOHs (50.5%) from 120 community pharmacies (Table 1). Three MOAs and nine MOHs were excluded due to incomplete or duplicate collection form. Seven students provided less than 20 prescriptions, which resulted in 63 missing medical orders. Family caregiver and other relatives had to bring the MO to the community pharmacy significantly more frequently for MOHs than from MOAs (n = 366, (31.3%) and n = 166 (14.4%), respectively; *p* < 0.001). Difficulty in prescriber identification was significantly more frequent for MOHs than for MOAs (4.8% versus 0.5%; *p* < 0.001).

Descriptions of DRPs and PIs and comparisons between MOA and MOH are shown in Table 2. A total of 169 DRPs were detected by CPs. DRPs were significantly more frequent for MOHs than for MOAs (n = 114 (9.7%) vs. n = 55 (4.8%); *p* < 0.001). There were 99 hospital prescriptions (8.4%) and 53 ambulatory prescriptions (4.6%) with at least one DRP. The most frequent DRPs were improper prescription (n = 61, 36.1%) and dosage problems (n = 42, 24.9%). There was no difference in DRP and PI subtype frequencies depending on the MO setting. The PI acceptance rate by the prescriber was 58.2% (MOAs) and 62.2% (MOHs), with no significant differences. Major or lethal potential clinical impacts tended to be more frequent for MOAs than for MOHs (n = 13 (24.5%) vs. n = 12 (12.4%); *p* = 0.056).

### 3.1. Impact on Patient Care Pathway

Unplanned consequences for patients were significantly more frequent for MOHs than for MOAs (7.4% vs. 3.0%; *p* < 0.001), mainly due to the delay for the initiation of a new drug (*p* < 0.001) (Table 3). Immediate nondispensed MOs were significantly more frequent for MOHs than for MOAs (20.6% vs. 6.3%; *p* < 0.001). In case of delayed dispensation, the dispensation occurred mainly within 24 h (80.8%) without differences between MOAs (75.0%) and MOHs (82.7%). Patient burden was assessed as minor by community pharmacists in most cases (82.2%) without differences between MOHs (86.1%) and MOAs (81.0%). For five (2.0%) MOHs, the impact was rated as major by the CP when the patient had to return to the pharmacy when there was a long distance between the CP and the patient’s home.

### 3.2. Impact on Community Pharmacy Workflow

The impact on community pharmacy workflow is presented in Table 3. Time-consuming activities > 30 min or disturbed CP team activities were more frequent for MOHs than for MOAs (20.3% vs. 8.3%; *p* < 0.001). Difficulties in contacting prescribers were significantly more frequent for MOHs than for MOAs (52.2% vs. 16.7%; *p* < 0.001) with an average of two or three interlocutors before obtaining the lacking information.

## 4. Discussion

Our study has limitations. Among them, the drug names involved in the DRPs and PIs were neither collected nor reviewed. Additionally, the significance of the interactions was only assessed by pharmacy students and their mentor. The investigators did not perform a secondary analysis on medical orders or pharmacist interventions. Moreover, clinical data were not collected during our study.

Nevertheless, to date, this study is the first to conduct a comparison of DRPs and PIs on MOs provided from both hospital and ambulatory settings considering clinical and organizational impact on patient care pathway and the workflow of the CP teams. Our study highlighted that MOs from hospital settings generate more PIs than medical orders from ambulatory settings. The students reported 8.3% and 4.6% of DRPs for MOs from hospital and ambulatory settings, respectively. This DRP rate detection is in line with previous studies that reported a DRP rate of 0.26% to 7% [11,12,13,14,15,16,17,18]. With nearly twice as many DRPs on MOHs than on MOAs, prescriptions from hospitals seem to cause more problems during the dispensing process. The same observation was reported in a Norwegian study also comparing hospital and ambulatory care prescriptions, with DRPs in 7.1% for MOHs versus 1.5% for MOAs [11].

The most frequently reported DRP is related to improper prescription, such as a lack of clarity or missing prescriber information. This category included regulatory admissibility, which was statistically lower for MOHs in our study. In practice, prescribers from ambulatory care have their name and licence number on their own prescription paper, unlike hospital prescribers (mostly medicine residents) using prescription papers displaying only the department’s name. This lack of identification information results in nonadmissibility or admissibility after a minor correction. Similar results were also found in another French study where the name of the physician was unspecified on one-third of the medical orders dispensed by the CPs [5]. Nevertheless, this problem should decrease with the emergence of computerized discharge orders. The frequencies of the two main DRPs showed no significant difference according to the setting and are in agreement with those reported in previous studies [5,11,19,20].

A major aspect of the dispensation process is information availability when needed. Two difficulties were highlighted for MOHs: Firstly, MOHs are more often brought by a relative or a professional caregiver (after hospital discharge) than by the patient. These third parties might lack information regarding the patient’s condition, history, and/or administrative information. Secondly, CPs reported difficulty in contacting the MOH prescriber. The same observation was reported by Trausch and Green in 2017 with at least two interlocutors for 48% of hospital calls [7]. Efforts are needed to improve the availability of patient data to CPs (e.g., by sharing electronic healthcare records out of the hospital setting). Despite these difficulties in reaching the prescribers, the acceptance rate of PIs by the prescriber or the patient for some minor DRPs was over 90%, which should be considered a good indicator of PI relevance in this study; PI acceptance rate in comparable studies ranged from 66% to 94% [6,19].

This study reported the potential clinical impact of PIs on patient care pathway and on CP workflow related to MOs from the hospital or ambulatory settings separately. For the potential clinical impact of PIs, a trend toward a higher number of PIs with major or lethal impact was found for MOAs than for MOHs. Although not reaching statistical significance, these results confirm that the more frequent occurrence of DRPs for MOHs is not correlated to their potential clinical impact.

For the patient care pathway, MOHs are associated with more unplanned consequences for the patient and nonimmediate-dispensed MOs, even for MOs without DRPs. We hypothesize that this finding is linked with unfrequently used and/or very expensive prescribed drugs such as treatments for rare disease conditions (orphan disease), including targeted therapies for cancers. In 2016, Michel et al. also reported the problem of delayed treatment initiation with 23.4% of DRPs occurring due to a drug being out of stock [4]. This delay can then result in noncompliance with the treatment or an additional visit to the pharmacy for the patient. The problem of out-of-stock drugs may be anticipated by having the hospital pharmacist perform a medication reconciliation at hospital discharge. This key step recently showed its benefits in terms of DRP exposure and severe iatrogenic problems [16].

The organizational consequences for CPs, mostly related to the time spent per MO, were also significantly different according to the setting. Nevertheless, time spent by the CP team was less than 15 min for the majority of MOs, which agrees with two previous studies that found extra time to complete the MOs to be less than 5 min [11,21]. As studies found that out-of-stock drugs are frequently involved in extended time before treatment initiation, one solution could be the optimization of the communication between hospital and community pharmacists. This system has been developed in the field of oral anticancer drugs by the implementation of pharmaceutical care [22].

Finally, our study highlights a lack of communication between hospital practitioners (including hospital pharmacists) and community pharmacists. Our results provide an accurate comparison between DRPs from ambulatory and from hospital settings, not only focusing on patients from the hospital. In 2017, the French Society of Clinical Pharmacy (SFPC) proposed a new model of clinical pharmacy for France, which is based on the implementation of personal pharmaceutical plans dedicated to management adapted to the patient’s pathway. It includes the performance of medication reconciliation at patient discharge by ensuring collaboration between hospital and community pharmacists. This process has not been systematically implemented for all patients (hospital discharge or post consultation) due to a lack of human resources. However, two French studies have highlighted the benefit of medication reconciliation at discharge [23,24]. In order to enhance the communication level without relying exclusively on the hospital pharmacist, one option is the implementation of the status of “corresponding pharmacist” that has been recently authorized by a French national law [25]. Given the recognition of this new status, community pharmacists could be assigned new competencies for adjustments or switches from one drug to another, which may limit the need of hospital contact. Moreover, recognition of the corresponding pharmacist by the hospital could allow the pharmacist to send all correspondence such as hospital consultation letters or hospital discharge letters, as was already claimed [26]. Some countries such as Ohio (United States of America), Canada, and United Kingdom have already implemented programs to enhance the transition in care with positive results on patients [27] and less hospital readmissions, respectively [28,29]. For a few months in the United Kingdom, a program named Transfers of Care Around Medicines (TCAM) uses an online platform to transfer the discharge information to the community pharmacy chosen by the patient, which can contact him for a follow-up [30]. An increase in such programmes is to be expected in future years to improve the coordination and continuity of outpatient care.

## 5. Conclusions

Most of the problems reported in this study, especially those related to the admissibility of MOHs, seem avoidable with the implementation of simple measures such as the use of assistive prescription software. Communication between community pharmacists, general practitioners, and hospital clinicians appears essential for the continuity of care and to make the dispensing process more fluid. The implementation of community and hospital pharmacist communication as a whole or a part of medication reconciliation at hospital discharge is probably the most accessible measure to implement, but whether resources are available remains questionable.

## Figures and Tables

**Table 1:** Description of the regulatory overview of the 2325 medical orders collected during the study period.

Variable ^a^	MOs	MOs from Ambulatory Setting	MOs from Hospital Setting	*p*-Value
	n = 2325	n = 1151	n= 1174	
**Individual bringing the MO to the community pharmacy**				
Patient	1683 (72.4)	938 (81.5)	745 (63.3)	*p* < 0.001
Family caregiver/relatives	532 (22.9)	166 (14.4)	366 (31.3)	
Professional caregiver	47 (2.0)	18 (1.6)	29 (2.5)	
Sent by email or fax	63 (2.7)	29 (2.5)	34 (2.9)	
**Status of the prescriber**				
Graduated physician	1977 (85.0)	1102 (95.8)	875 (74.4)	*p* < 0.001
Medicine resident	152 (6.5)	6 (0.5)	146 (12.4)	
Other ^b^	40 (1.7)	20 (1.7)	20 (1.7)	
Nonidentified	75 (3.2)	1 (0.1)	74 (6.3)	
Discrepancy ^c^	83 (3.6)	22 (1.9)	61 (5.2)	
**Prescriber identification: identity (surname)**				
Easy to identify	2263 (97.3)	1145 (99.5)	1118 (95.4)	*p* < 0.001
Hard to identify	34 (1.5)	4 (0.3)	30 (2.6)	
Not possible to identify	28 (1.2)	2 (0.2)	26 (2.2)	

MO: medical order; n: number; ^a^ results presented as number (%); ^b^ other authorized health professional: nurses, dentist, kinesiologist, or midwife; ^c^ discrepancy between the header in the prescription form and signature; ^d^ comparison between non-identified prescriber, discrepancy, and other prescriber statuses. The bracket symbol shows the variables involved in the statistical test and the *p*-value.

**Table 2:** Description of the 169 DRPs and PIs from 152 MOs according to the French Society of Clinical Pharmacy and the CLEO Tool.

Variable ^a^	DRPs on All MOs (n = 169)	DRPs on MOs from Ambulatory Setting (n = 55)	DRPs on MOs from Hospital Setting (n = 114)
Drug-related problem			
Improper prescription	61 (36.1)	11 (20.0)	50 (43.9)
Dosage problem	42 (24.9)	15 (27.3)	27 (23.7)
Drug interaction	11 (6.5)	6 (10.9)	5 (4.4)
Drug or medical device not received by the patient	15 (8.9)	6 (10.9)	9 (7.9)
Drug omission	13 (7.7)	6 (10.9)	7 (6.1)
Contraindication/nonconformity to guidelines	9 (5.3)	5 (9.1)	4 (3.5)
Therapeutic redundancy	9 (5.3)	2 (3.6)	7 (6.1)
Unjustified drug prescription	7 (4.1)	3 (5.5)	4 (3.5)
Adverse drug reaction	2 (1.2)	1 (1.8)	1 (0.9)
Pharmacist intervention			
Dose adjustment	47 (27.8)	16 (29.1)	31 (27.3)
Optimization of the dispensing/administration mode	43 (25.4)	10 (18.2)	33 (29.0)
Discontinuation or refusal to deliver	30 (17.8)	9 (16.4)	21 (18.5)
Drug switch	25 (14.8)	14 (25.4)	11 (9.6)
Addition of a new drug	18 (10.7)	6 (10.9)	12 (10.4)
Choice of administration route	3 (1.8)	0	3 (2.6)
Drug monitoring	3 (1.8)	0	3 (2.6)
Intervention follow-up			
Accepted by the prescriber	103 (60.9)	32 (58.2)	71 (62.2)
Not accepted by the prescriber with no justification	1 (0.6)	1 (1.8)	0
Not accepted by the prescriber with justification	2 (1.2)	0	2 (1.8)
Refusal to deliver, with a phone call to the prescriber	3 (1.8)	1 (1.8)	2 (1.8)
Refusal to deliver with no call to the prescriber	3 (1.8)	3 (5.5)	0
Accepted by the patient (the prescriber is not contacted)	55 (32.5)	17 (30.9)	38 (33.3)
Not accepted by the patient	2 (1.2)	1 (1.8)	1 (0.9)
Clinical impact ^b^			
Harmful	9 (5.3)	1 (1.8)	8 (7.0)
Null	43 (25.4)	14 (25.5)	29 (25.4)
Minor	51 (30.2)	18 (32.7)	33 (28.9)
Moderate	22 (13.0)	7 (12.7)	15 (13.2)
Major	20 (11.8)	10 (18.2)	10 (8.8)
Lethal	5 (3.0)	3 (5.5)	2 (1.8)
No determined ^c^	19 (11.2)	2 (3.6)	17 (14.9)

n: number; ^a^ Results presented as number (%), ^b^ according to the CLEO tool: Clinical, Economic and Organizational; ^c^ not completed by the community pharmacist.

**Table 3:** Impact of medical orders from ambulatory and hospital settings on patient care pathway and on community pharmacy workflow.

All Medical Orders	MOs	MOs from Ambulatory Setting	MOs from Hospital Setting	*p*-Value
	n = 2325	n = 1151	n = 1174	
Impact on community pharmacy workflow				
Time spent per MO for community pharmacy team				
Minor (<15 min)	1992 (85.7)	1056 (91.7)	936 (79.7)	*p* < 0.001
Moderate (15–30 min)	284 (12.2)	87 (7.6)	197 (16.8)	
Major (>30 min or disturbance of CP workflow ^a^)	49 (2.1)	8 (0.7)	41 (3.5)	
Impact on community on patient care pathway				
Clinical outcome on patient pathway				
No unplanned consequence	2203 (94.8)	1116 (97.0)	1087 (92.6)	*p* < 0.001
Unplanned consequence:	122 (5.2)	35 (3.0)	87 (7.4)	
- Delay in treatment initiation ^b^	76 (3.3)	21 (1.8)	55 (4.7)	
- Treatment interruption ^c^	7 (0.3)	1 (<0.1)	6 (0.5)	*p* = 0.63
- Referral to the GP/attending physician	7 (0.3)	2 (0.2)	5 (0.4)	
- Other	32 (1.4)	11 (1.0)	21 (1.8)	
Non immediate dispensed MO	314 (13.5)	72 (6.3)	242 (20.6)	*p* < 0.001
Delay for availability of medicine in case of nonimmediate dispensing				
In the half-day	116 (36.9)	28 (38.9)	88 (36.4)	*p* = 0.21
Within 24 h	138 (43.9)	26 (36.1)	112 (46.3)	
Beyond 24 h	60 (19.1)	18 (25.0)	42 (17.3)	
Patient burden in case of non-immediate dispensed MO				
Minor ^d^	258 (82.2)	62 (86.1)	196 (81.0)	*p* = 0.32
Moderate ^e^	51 (16.2)	10 (13.9)	41 (17.0)	
Major ^f^	5 (1.6)	0	5 (2.0)	

n: number; ^a^ For example, unplanned necessity for community pharmacist to travel another pharmacy/supplier for medicine supply; ^b^ delay in the initiation of a medicine (e.g., because of unavailability, lack of information, etc.); ^c^ interruption of medicine consumption (e.g., from hospital to ambulatory setting) because of the unavailability of medicine, for example; ^d^ minimal burden (community pharmacy close to patient home or mobile patient), ^e^ Moderate burden (CP distant from the patient home and/or less mobile patient); ^f^ Major burden (CP distant from patient home and immobile patient). The bracket symbol shows the variables involved in the statistical test and the *p*-value. “-“ is to distinguish the subcategories of unplanned consequence.

## Data Availability

The data presented in this study are available on request from the corresponding author. The data are not publicly available due to privacy.

## Supplementary Materials

The following are available online at https://www.mdpi.com/article/10.3390/pharmacy10010002/s1, File S1: Information collection form used at the community pharmacy, File S2: ORDHOSPIVILLE study design flow chart.
