# Supplementary material for: Clinical and Organizational Impacts of Medical Ordering Settings on Patient Pathway and Community Pharmacy Dispensing Process: The Prospective ORDHOSPIVILLE Study
Source: Pharmacy (Basel). 2021 Dec 23;10(1):2. doi: 10.3390/pharmacy10010002 (PMC8788414; doi:10.3390/pharmacy10010002)
Supplement: Supplementary file 1 [file pharmacy-10-00002-s001.zip › pharmacy-1471428-supplementary/Supplementary File S1.pdf]

Form n°:

Information collection form at the community pharmacy

PART 1: Information about medical order

Date of medical order: ……………………………………..
Date and time of presentation of medical order at the community pharmacy: …………………

Characteristics of medical order:
☐ Medical order from hospital setting (public or private health establishment)
☐ Medical order from ambulatory setting

If medical order from hospital, does it come from:
☐ Out-patient service ☐ Hospital discharge medical order ☐ I do not know

Reglementary admissibility of medical order:
☐ Immediately admitted ☐ Admitted after minor correction ☐ Not admitted
If not admitted, specify: ☐ Incorrect medical order support
☐ Not allowed prescriber
☐ Other:

Nature of signatory prescriber:
☐ Graduated physician with licence number ☐ Medicine resident with licence number
☐ Other qualified prescriber (midwife, nurse, physiotherapist) ☐ Non identified
☐ Discrepancy between the header and the signature

Prescriber identification **(1)**:

Surname:	☐ Easy to identify	☐ Hard to identify	☐ Not possible to identify
Firt name:	☐ Easy to identify	☐ Hard to identify	☐ Not possible to identify
Qusalification:	☐ Easy to identify	☐ Hard to identify	☐ Not possible to identify
Licence number:	☐ Easy to identify	☐ Hard to identify	☐ Not possible to identify

Individual bringing the prescription:

☐ Patient ☐ Professional caregiver (healthcare provider, nurse, other)
☐ Family caregiver / relatives ☐ Medical order sent by fax, email…

Lines of medical order:
Number of line present on medical order (drug and other health product) **(2)**: …….
Number of drug: …….
Number of health product except drug: …….

Need to contact the prescriber:
☐ Yes ☐ No
If yes, specify the degree of difficulty to contact him: Very easy - Easy - Difficult - Very difficult
- How many contacts before obtaining information: ……
- Who provide information (several interlocutors possible):

☐ The prescriber ☐ An other qualified contact (other doctor or nurse)
☐ No qualified contact ☐ Impossible to contact

PARTS 2 and 3: Fill in only if necessity to contact the prescriber

PART 2: Pharmacist intervention

Pharmacist intervention performed:
☐ Yes ☐ No
If yes, number of pharmacist intervention performed on medical order:
For each pharmacist intervention, fill in the dedicated form in appendix (PI form from SFPC)

Clinical impact of pharmacist intervention according to the CLEO tool **(3)**
In case of pharmacist intervention performed, please rate the degree of impact on the patient's health status according to your point of view:
☐ Harmful ☐ Null ☐ Minor ☐ Moderate ☐ Major ☐ Lethal
☐ Non determined

PART 3: Assessment of pharmaceutical care

Time of availability of health product(s) in case of non-immediate dispensing (Impossible to contact the prescriber and/or necessity of supply and/or different location of dispensation/hospital pharmacy…) **(4)**

☐ Within the half day ☐ Within 24 hours ☐ Beyond 24 hours

Organizational outcomes:

- Patient burden in case of non-immediate dispensed MO **(5)**:
  ☐ Minor ☐ Moderate ☐ Major

- Consequence of the time spent for the community pharmacy team (cumulated time):
  ☐ Minor (less than 15 min)
  ☐ Moderate (between 15 and 30 min)
  ☐ Major (more than 30 min or unplanned physical movement for a member of community pharmacist team)

- Outcome on patient’s care pathway:
  ☐ Delay in the patient’s care pathway
  ☐ Treatment interruption
  ☐ Redirection to general practitioner
  ☐ None. Specify: ……………………………………………………………………
  ☐ Other. Specify: ……………………………………………………………………...

Easy-to-use study procedure

When a medical order is included in the study, it must be copied anonymously and transmitted with the information collection form and the PI forms form SFPC if pharmacist intervention is required.

(1) : Choose the box « easy to identify » when the information is clear; « hard to identify» if the information is not correctly visible, incomplete or difficult to locate on the medical order

(2) : Medical devices (bandages…), phytotherapy product, homeopathy

(3) : SPFC reference

1. Clinical impact

Score	Impact	Definition: The clinical impact is evaluated according to the most likely case expected, not the worst/best case
-1C	Nuisible	The PI can lead to adverse outcomes on clinical status, knowledge, satisfaction, patient adherence and/or quality of life of the patient.
0C	Null	The PI can have no influence on the patient regarding the clinical status, knowledge, satisfaction, patient adherence and or quality of life of the patient.
1C	Minor	The PI can improve knowledge, satisfaction, medication adherence and/or quality of life OR the PI can prevent damage that does not require monitoring/treatment.
2C	Moderate	The PI can prevent harm that requires further monitoring/treatment, but does not lead or do not extend a hospital stay of the patient.
3C	Major	The PI can prevent harm which causes or lengthens a hospital stay OR causes permanent disability or handicap.
4C	Lethal	The PI can prevent an accident that causes a potentially intensive care or death of the patient.
ND	Non-determined	The available information does not determine the clinical impact.

- The clinical impact is evaluated for the patient's benefit.
- Harm: alteration of the physical and mental capacities arising from an accident or illness.
- Quality of life: physical function (autonomy, physical abilities, capacity to perform the tasks of daily life ...), psychological (anxiety, depression, emotion ...), social (relative to family environment, friendly or professional, engaging in personal relationships, participation in social and leisure activities ...) and somatic (symptoms related to the disease).
- Monitoring: monitoring clinically relevant (physiological or psychological), biological.
- Treatment: changing therapy or adding an additional medical / surgical treatment.

(4) : If several answers possible, indicate the longest time

(5) : Patient burden in cas of non-immediate dispensed MO

- Minor burden: community pharmacy close to patient home or mobile patient
- Moderate burden: CP distant form the patient home and/or less mobile patient
- Major burden: CP distant from patient home and immobile patient
