# Supplementary material for: Clinical and Organizational Impacts of Medical Ordering Settings on Patient Pathway and Community Pharmacy Dispensing Process: The Prospective ORDHOSPIVILLE Study
Source: Pharmacy (Basel). 2021 Dec 23;10(1):2. doi: 10.3390/pharmacy10010002 (PMC8788414; doi:10.3390/pharmacy10010002)
Supplement: Supplementary file 1 [file pharmacy-10-00002-s001.zip › pharmacy-1471428-supplementary/Supplementary File S2.pdf]

ORDHOSPIVILLE study design – Flow Chart

1 Select the medical orders and scan them
(By alternating 1 medical order from hospital setting then 1 medical order from ambulatory setting)

How to select medical order from hospital setting: As you encounter them during your internship: take every medical order (and not only if a problem is detected) corresponding to inclusion criteria:
- Medical order concerning every drug and/or medical device and/or any other health product.
- A medical order is qualified as « from hospital setting » if it comes from a hospital discharge, or a hospital consultation in private or public health institution

- Every community pharmacy has to included **10** medical orders from hospital setting
- Inclusion is made by medical order (for example: one patient can have 4 medical orders from hospital setting, in this case, we count 4 inclusions)

How to select medical order from ambulatory setting:
- Alternate the medical orders: when you included a medical order from hospital setting, you select the following medical order from ambulatory setting (with drug and/or medical device and/or any other health product)
- A medical order is qualified as « from ambulatory setting » if it does not come from hospital.
- Every community pharmacy must include **10** medical orders from ambulatory setting

Particular case:
If you include 3 medical orders from hospital setting from the same patient, you will then include the 3 following medical order from ambulatory setting coming from any patient you will meet.

2 Fill in the online questionnaire
(1 questionnaire by medical order)

How to fill in the questionnaire:
- Fill it in as soon as possible so as not to lose any information, but without interfering with the filling of the medical order
- For the "clinical impact" part, a procedure is available online
- Scan the ANONYMISED medical order and integrate it into the collection form
- Include the pharmacist intervention form(s) if applicable
